# Supplementary material for: The zinc metalloprotein MigC impacts cell wall biogenesis through interactions with an essential Mur ligase in Acinetobacter baumannii
Source: PLoS Pathog. 2025 Jun 16;21(6):e1013209. doi: 10.1371/journal.ppat.1013209 (PMC12208494; doi:10.1371/journal.ppat.1013209)
Supplement: S1 Fig — Ribbon diagram of AlphaFold3 model of A1S_0934 with one Zn2+ ion bound, showing the G-domain (pale blue), C-terminal domain (grey), and the CxCC motif (inset) composed of C71, C73, and C74. Regions of conserved residues identified by SSN are denoted as G1 (cyan), G2 Switch I (orange), G3 Switch II (purple), G4 (royal blue), and G5 (yellow). The position of the highly conserved aspartic acid (D) to glutamic acid (E) substitution in the G3 loop (E99) of the protein is identified by the arrow. (PDF) [file ppat.1013209.s001.pdf]

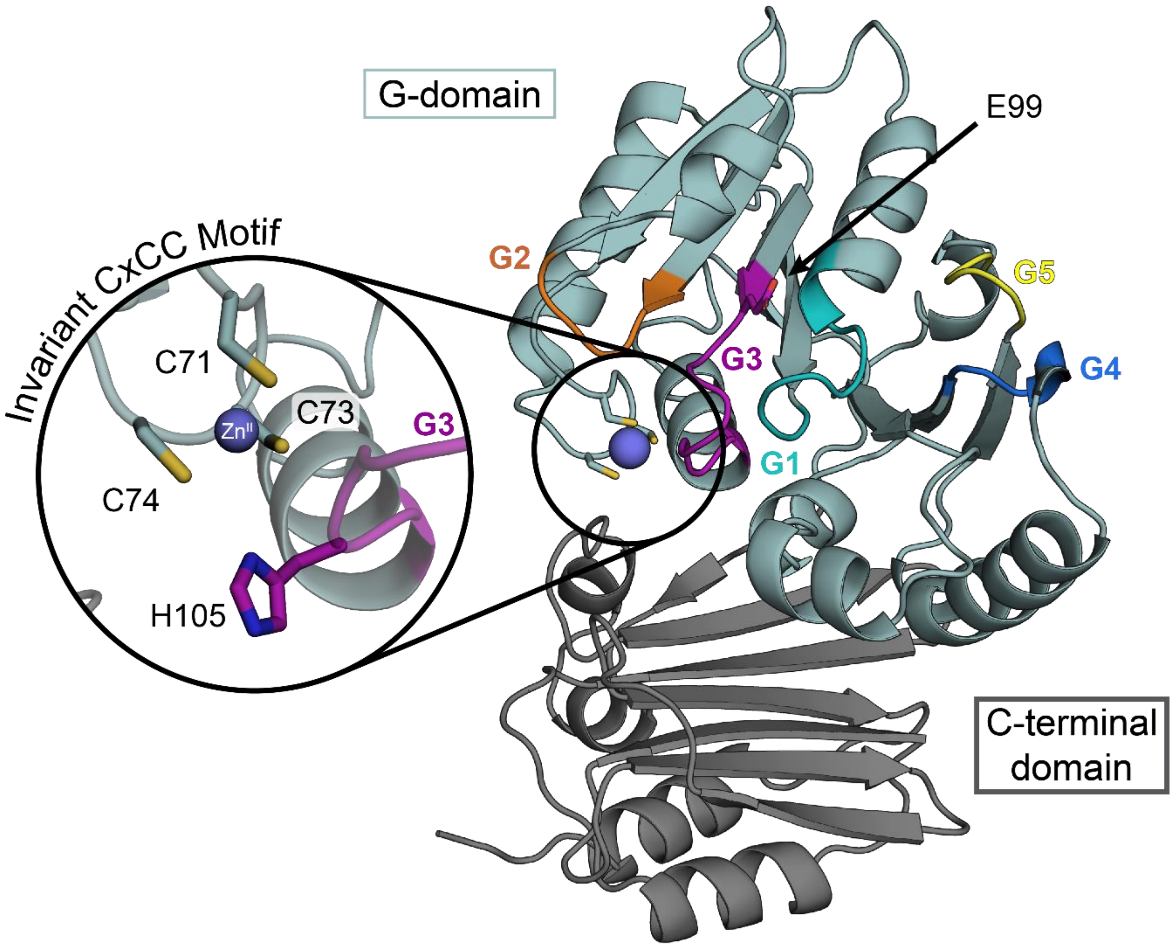

<u>Supplementary Figure 1</u>: AlphaFold3 Model of A1S_0934 predicts global structural changes upon nucleotide binding. Ribbon diagram of AlphaFold3 model of A1S_0934 with one Zn^{2+} ion bound, showing the G-domain (pale blue), C-terminal domain (grey), and the CxCC motif (inset) composed of C71, C73, and C74. Regions of conserved residues identified by SSN are denoted as G1 (cyan), G2 Switch I (orange), G3 Switch II (purple), G4 (royal blue), and G5 (yellow). The position of the highly conserved aspartic acid (D) to glutamic acid (E) substitution in the G3 loop (E99) of the protein is identified by the arrow.
