# Supplementary material for: The zinc metalloprotein MigC impacts cell wall biogenesis through interactions with an essential Mur ligase in Acinetobacter baumannii
Source: PLoS Pathog. 2025 Jun 16;21(6):e1013209. doi: 10.1371/journal.ppat.1013209 (PMC12208494; doi:10.1371/journal.ppat.1013209)
Supplement: S2 Fig — Transmission electron microscopy was performed on WT or Δ0934 cells in LB ± 40 μM TPEN. Cells were further assessed for (A) cell envelope, (B) inner, and (C) outer membrane width using ImageJ software. *p < 0.05 by one-way ANOVA. (PDF) [file ppat.1013209.s002.pdf]

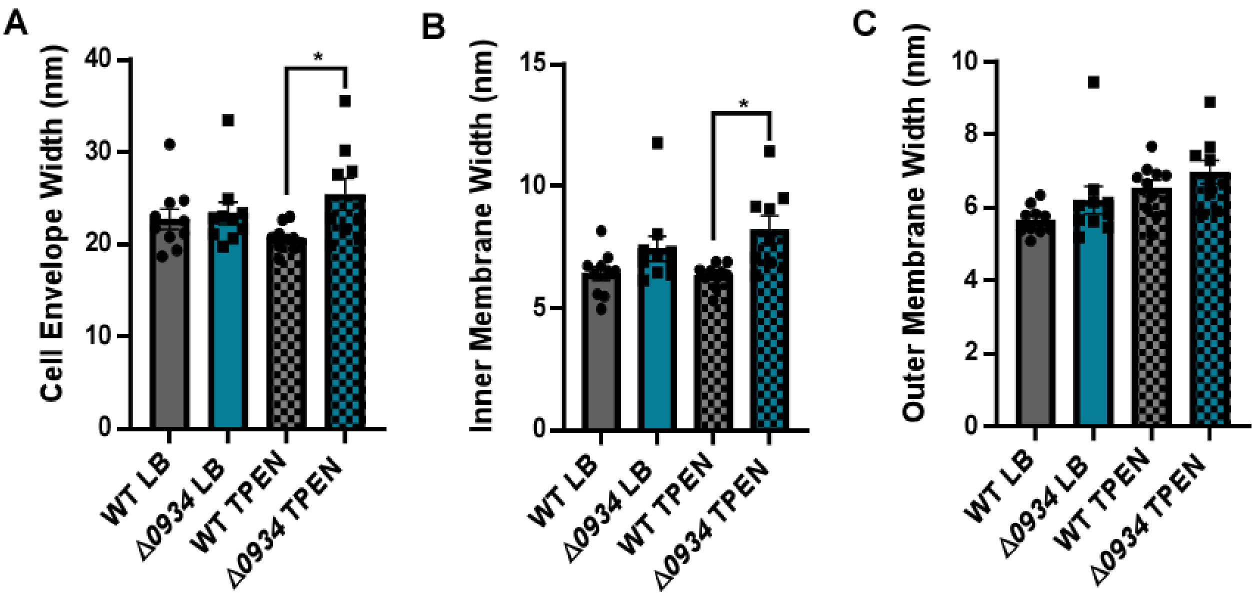

Supplementary Figure 2: A1S_0934 function may be affected by multiple divalent cations, which contributes to an elongated cell morphology. Transmission electron microscopy was performed on WT or Δ*0934* cells in LB ± 40 μM TPEN. Cells were further assessed for (**A**) cell envelope, (**B**) inner, and (**C**) outer membrane width using ImageJ software. *$p<0.05$ by one-way ANOVA.
