# Supplementary material for: The zinc metalloprotein MigC impacts cell wall biogenesis through interactions with an essential Mur ligase in Acinetobacter baumannii
Source: PLoS Pathog. 2025 Jun 16;21(6):e1013209. doi: 10.1371/journal.ppat.1013209 (PMC12208494; doi:10.1371/journal.ppat.1013209)
Supplement: S3 Fig — (A) 1-by-1 interaction domain mapping yeast two-hybrid performed between full-length MigC and a truncated form of MurD, AA55–354, by Hybrigenics. Growth on agar with histidine supplemented indicates interactions between MigC and MurD. (B) Inhibition curves of MurD activity with varied apo MigC with (red) and without GTP (grey), Zn loaded MigC with GTP (blue), Zn loaded E99A MigC with GTP. A global curve fitting was performed to determine maximum inhibition of MurD at saturating MigC to be 75%. Curve fittings determined Ki for apo MigC without GTP to be 20 ± 3 µM, Ki for apo MigC with GTP to be 43 ± 7 µM, Ki for Zn MigC with GTP to be 32 ± 6 µM, and Ki for Zn E99A MigC with GTP to be 21 ± 6 µM. (PDF) [file ppat.1013209.s003.pdf]

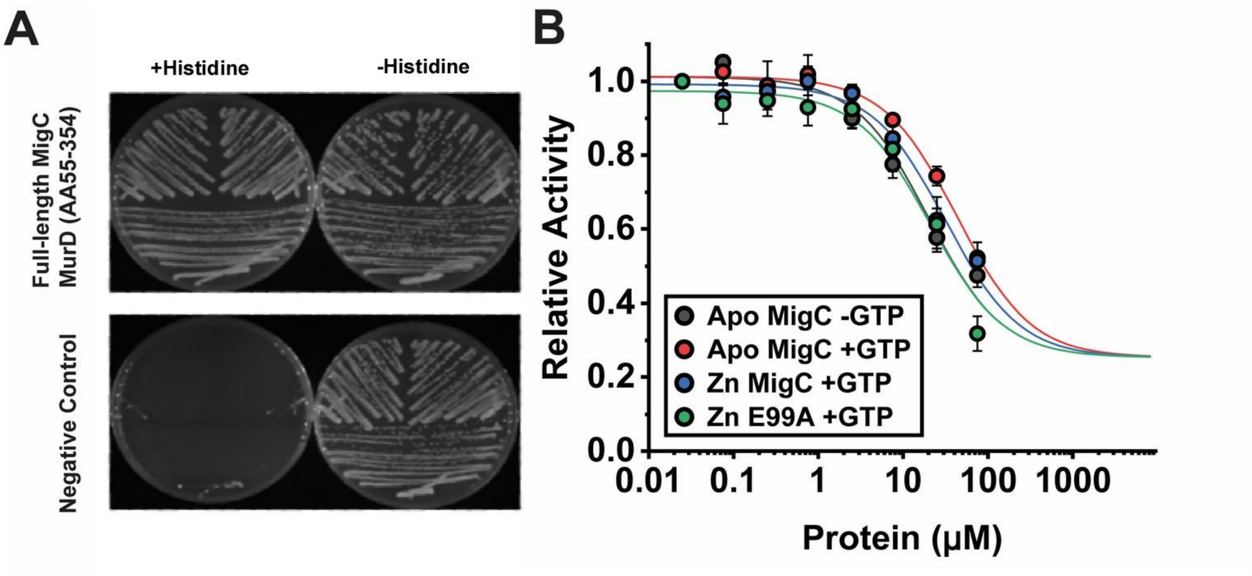

<u>Supplementary Figure 3</u>: MigC inhibits MurD activity under a variety of conditions. (A) A 1-by-1 interaction domain mapping yeast two-hybrid performed between full-length MigC and a truncated form of MurD, AA55-354, by Hybrigenics. Growth on agar with histidine supplemented indicates interactions between MigC and MurD. **(B)** Inhibition curves of MurD activity with varied apo-MigC with (red) and without GTP (grey), Zn loaded MigC with GTP (blue), Zn loaded E99A MigC with GTP. A global curve fitting was performed to determine maximum inhibition of MurD at saturating MigC to be 75%. Curve fittings determined K_i for apo-MigC without GTP to be 20 ± 3 μM, K_i for apo-MigC with GTP to be 43 ± 7 μM, K_i for Zn-MigC with GTP to be 32 ± 6 μM, and K_i for Zn-E99A MigC with GTP to be 21 ± 6 μM.
