# Supplementary material for: The zinc metalloprotein MigC impacts cell wall biogenesis through interactions with an essential Mur ligase in Acinetobacter baumannii
Source: PLoS Pathog. 2025 Jun 16;21(6):e1013209. doi: 10.1371/journal.ppat.1013209 (PMC12208494; doi:10.1371/journal.ppat.1013209)
Supplement: S4 Fig — (A) WT attTn7::dcas9 + pyde007::EV, and ΔmigC attTn7::Ptet + pyde007::EV strains were grown in LB ± 50, 100, and 150 ng/mL of AhTc with OD600 monitored over time. (B) WT attTn7::Ptet- + pYDE007-sgRNAmurD were grown in LB ± 50, 100, and 150 ng/mL of AhTc with OD600 monitored over time. (C) ΔmigC attTn7::Ptet dcas9 + pYDE007-sgRNAmurD were grown in LB ± 50, 100, and 150 ng/mL of AhTc with OD600 monitored over time. (D) WT attTn7::Ptet-dcas9 + pYDE007 (EV), ΔmigC attTn7::Ptet-dcas9 + pYDE007 (EV), WT attTn7::Ptet-dcas9 + pYDE007-sgRNAmurD, and ΔmigC attTn7::Ptet-dcas9 + pYDE007-sgRNAmurD ± 100 ng/mL AhTc were grown with OD600 monitored over time. (E) These same strains were grown ± 50 ng/mL AhTc ± 20 μM TPEN with OD600 monitored over time. (PDF) [file ppat.1013209.s004.pdf]

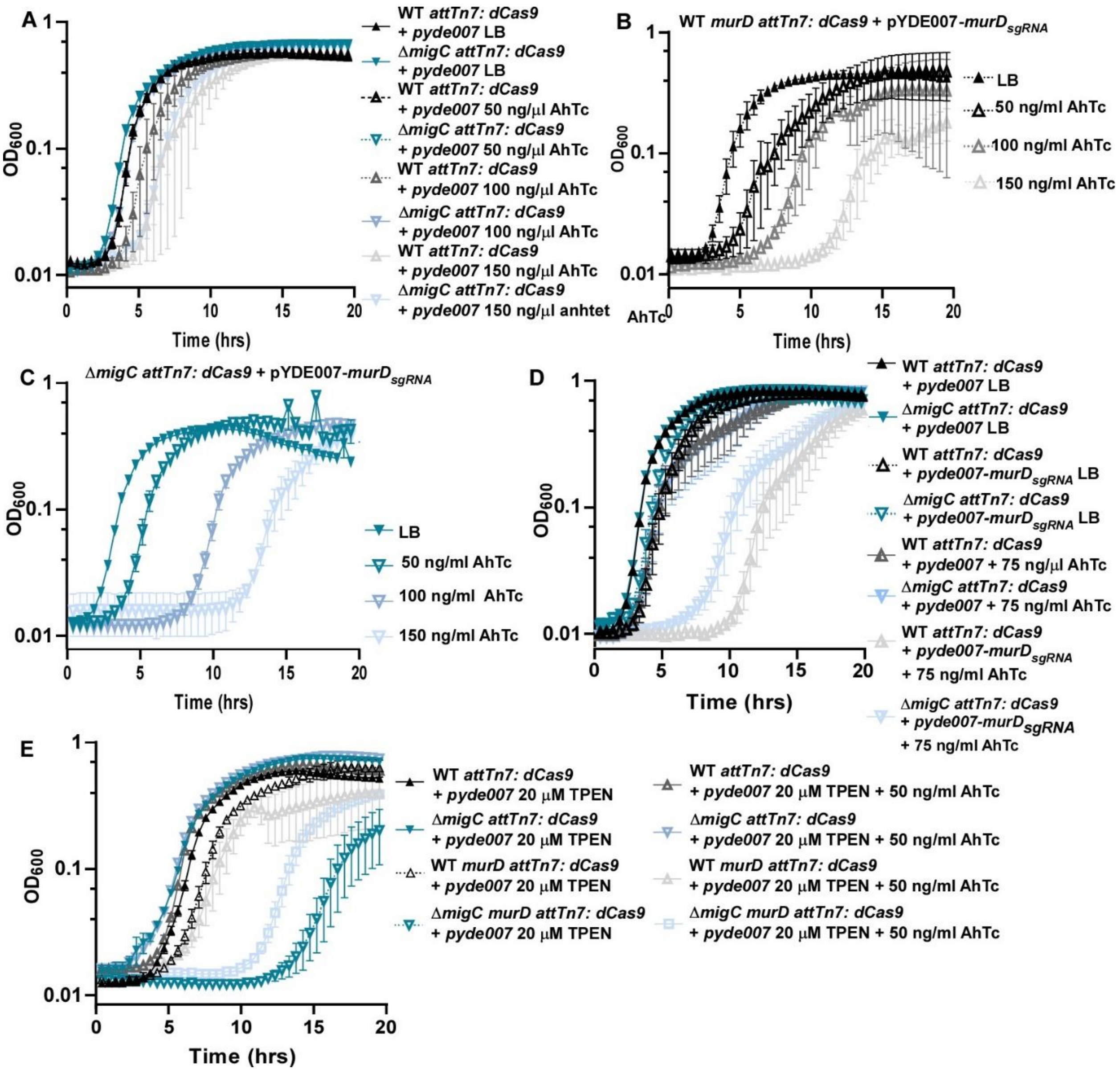

<u>Supplementary Figure 4</u>: **Knocking down or overexpressing *murD* is detrimental for *A. baumannii* growth.** (**A**) WT *attTn7::dcas9* + *pyde007::EV*, and Δ*migC attTn7::P$_{tet}$*- + *pyde007::EV* strains were grown in LB ± 50, 100, and 150 ng/mL of AhTc with OD_{600} monitored over time. (**B**) WT *attTn7::P$_{tet}$*- + pYDE007-sgRNA$_{murD}$ were grown in LB ± 50, 100, and 150 ng/mL of AhTc with OD_{600} monitored over time. (**C**) Δ*migC attTn7::P$_{tet}$*-*dcas9* + pYDE007-sgRNA$_{murD}$ were grown in LB ± 50, 100, and 150 ng/mL of AhTc with

OD_{600} monitored over time. (**D**) WT *attTn7::P$_{tet}$-dcas9* + pYDE007 (EV) , Δ*migC attTn7::P$_{tet}$-dcas9* + pYDE007 (EV), WT *attTn7::P$_{tet}$-dcas9* + pYDE007-sgRNA$_{murD}$, and Δ*migC attTn7::P$_{tet}$-dcas9* + pYDE007-sgRNA$_{murD}$ ± 100 ng/mL AhTc were grown with OD_{600} monitored over time. (**E**) These same strains were grown ± 50 ng/mL AhTc± 20 mM TPEN with OD_{600} monitored over time.
