# Supplementary material for: The zinc metalloprotein MigC impacts cell wall biogenesis through interactions with an essential Mur ligase in Acinetobacter baumannii
Source: PLoS Pathog. 2025 Jun 16;21(6):e1013209. doi: 10.1371/journal.ppat.1013209 (PMC12208494; doi:10.1371/journal.ppat.1013209)
Supplement: S5 Fig — (A) WT and ΔmigC integration controls and the complementation strain were grown for 20 hours in LB ± 7.5 μg/ml of CRO with OD600 monitored over time. Data are represented as percent of LB growth at 8 hours. (B-D) Mice were intranasally infected with WT or ΔmigC in mice given normal chow. Bacterial burdens were assessed at 36 hpi in the (B) kidney, (C) liver, or (D) spleen. Data are represented as the mean ± SEM with each point indicating the bacterial burdens from an individual mouse in a specific organ. The limit of detection is indicated as LOD. ***p < 0.001 by unpaired t test. (PDF) [file ppat.1013209.s005.pdf]

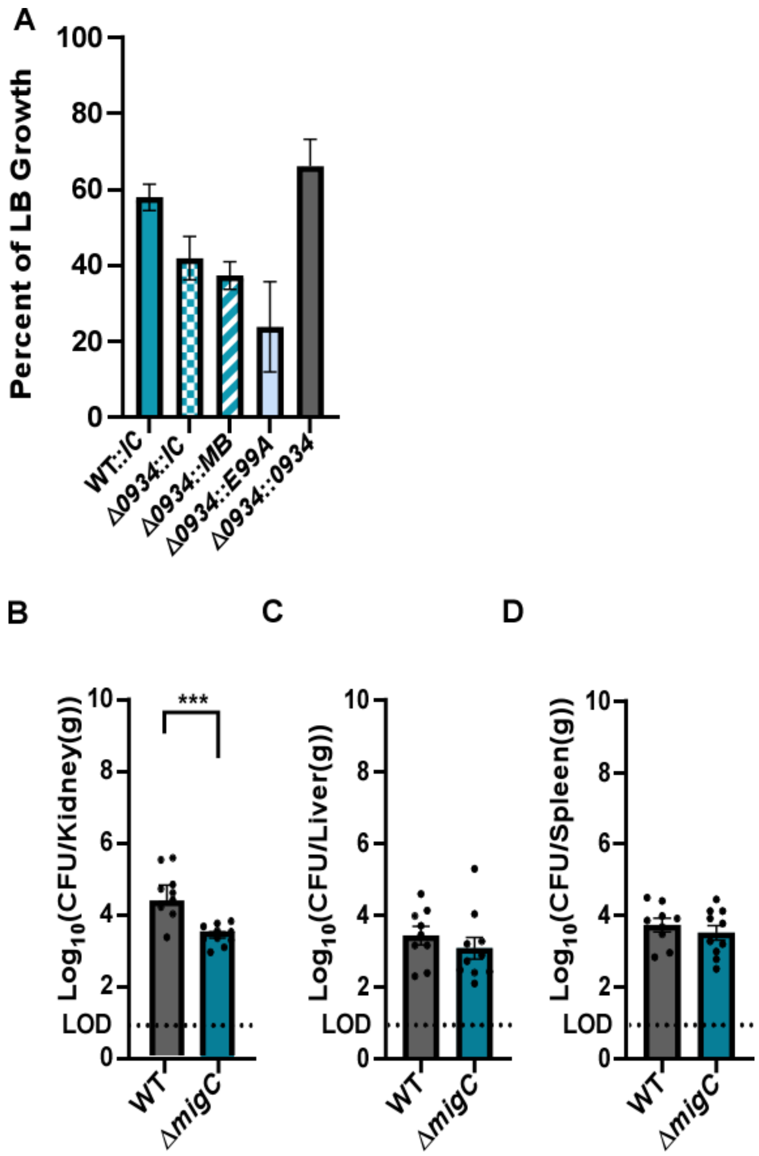

<u>Supplementary Figure 5</u>: MigC contributes to *A. baumannii* CRO-susceptibility and virulence. (**A**) WT and Δ*migC* integration controls and the complementation strain were grown for 20 hours in LB ± 7.5 µg/ml of CRO with OD_{600} monitored over time. Data are represented as percent of LB growth at 8 hours. (**B-D**) Mice were intranasally infected with WT or Δ*migC* in mice given normal chow. Bacterial burdens were assessed at 36 hpi in the (**B**) kidney, (**C**) liver, or (**D**) spleen. Data are represented as the mean ± SEM with each point indicating the bacterial burdens from an individual mouse in a specific organ. The limit of detection is indicated as LOD. ***p<0.001 by unpaired *t* test.
