# Supplementary material for: The zinc metalloprotein MigC impacts cell wall biogenesis through interactions with an essential Mur ligase in Acinetobacter baumannii
Source: PLoS Pathog. 2025 Jun 16;21(6):e1013209. doi: 10.1371/journal.ppat.1013209 (PMC12208494; doi:10.1371/journal.ppat.1013209)
Supplement: S6 Fig — (A) HPLC chromatograms of representative reactions without MurD (black), with MurD (light grey), and with MurD and MigC (blue). (B) Chemical standards of GDP (black), ADP (dark grey), ATP (light grey), UMA (cyan), and UMAG (blue) used to identify peaks in MurD reaction chromatograms. (PDF) [file ppat.1013209.s006.pdf]

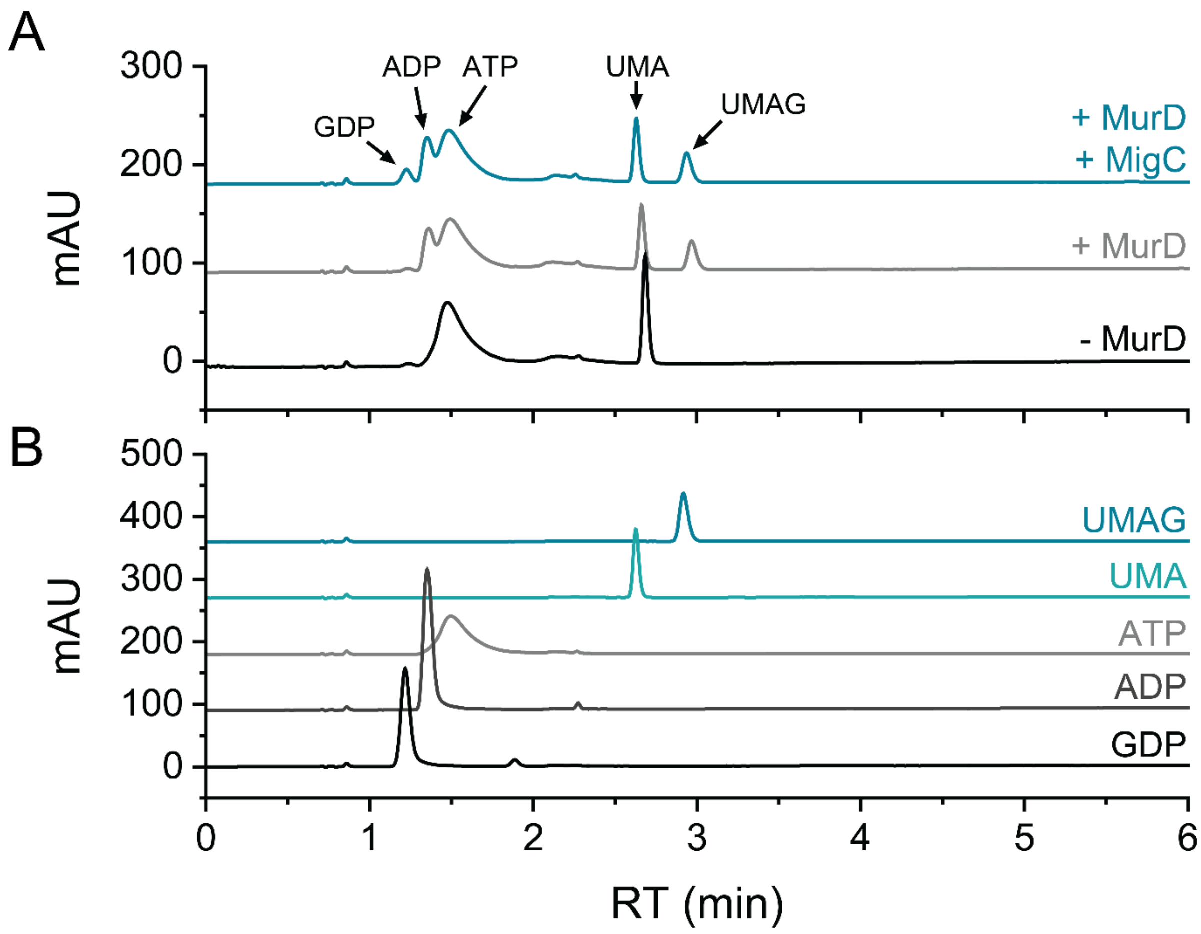

<u>Supplementary Figure 6</u>: MurD reaction specificity remains unchanged by MigC. (**A**) HPLC chromatograms of representative reactions without MurD (black), with MurD (light grey), and with MurD and MigC (blue). (**B**) Chemical standards of GDP (black), ADP (dark grey), ATP (light grey), UMA (cyan), and UMAG (blue) used to identify peaks in MurD reaction chromatograms.
