# Supplementary material for: The zinc metalloprotein MigC impacts cell wall biogenesis through interactions with an essential Mur ligase in Acinetobacter baumannii
Source: PLoS Pathog. 2025 Jun 16;21(6):e1013209. doi: 10.1371/journal.ppat.1013209 (PMC12208494; doi:10.1371/journal.ppat.1013209)
Supplement: S1 Table — aData were fitted only over a k-range of 1–12.6 Å due to monochromator imperfections. bIndependent metal-ligand scattering environment. cScattering atoms: C (carbon), N (nitrogen) and O (oxygen). dAverage metal-ligand bond length. eAverage metal-ligand coordination number. fAverage Debye-Waller factor in Å2 x 103. gNumber of degrees of freedom weighted mean square deviation between data and fit. (PDF) [file ppat.1013209.s007.pdf]

**<u>Supplementary Table 1</u>: Best-fit simulated parameters from the analysis of the Zn K-edge raw EXAFS for $Zn^{II}$-MigC**

| Sample[a] | Nearest Neighbor Ligand Environment[b] | | | | Long-Range Ligand Environment[c] | | | | |
|---|---|---|---|---|---|---|---|---|---|
| | Atom[c] | R(Å)[d] | C.N.[e] | $\sigma^2$[f] | Atom[c] | R(Å)[d] | C.N.[e] | $\sigma^2$[f] | $F'$[g] |
| $Zn^{II}$-MigC | S | 2.32 | 3.0 | 3.90 | C | 3.36 | 0.5 | 5.71 | 0.12 |
| (150 mM NaBr) | O/N | 2.05 | 1.0 | 5.04 | C | 4.02 | 1.0 | 4.22 | |
| $Zn^{II}$-MigC | S | 2.32 | 3.0 | 4.11 | C | 3.34 | 1.0 | 2.86 | 0.14 |
| (150 mM NaCl) | O/N | 2.06 | 1.0 | 5.61 | C | 4.05 | 2.0 | 3.30 | |

[a]Data were fitted only over a k-range of 1–12.6 Å due to monochromator imperfections.

[b]Independent metal-ligand scattering environment.

[c]Scattering atoms: C (carbon), N (nitrogen) and O (oxygen).

[d]Average metal-ligand bond length.

[e]Average metal-ligand coordination number.

[f]Average Debye-Waller factor in $Å^2 \times 10^3$.

[g]Number of degrees of freedom weighted mean square deviation between data and fit.
