# Supplementary material for: The zinc metalloprotein MigC impacts cell wall biogenesis through interactions with an essential Mur ligase in Acinetobacter baumannii
Source: PLoS Pathog. 2025 Jun 16;21(6):e1013209. doi: 10.1371/journal.ppat.1013209 (PMC12208494; doi:10.1371/journal.ppat.1013209)
Supplement: S2 Table — (PDF) [file ppat.1013209.s008.pdf]

<u>Supplementary Table 2</u>**: Strains used in this study.**

**Strain**	**Characteristics**	**Source**
Acinetobacter baumannii ATCC 17978VU	Wildtype	ATCC (Wijers *et al*., 2021) [74]
Escherichia coli DH5α		Lab stock
Escherichia coli pUC18-K1	Plasmid containing *aphA* gene	(Ménard *et al*., 1993) [75]
Escherichia coli DH5a pFLp2-Δ*migC::aphA*	*A1S_0934* (Δ*migC::aphA*) knockout vector	This study
Acinetobacter baumannii ATCC 17978VU Δ*migC*	Δ*migC::aphA*	This study
Escherichia coli DH5α pFLp2	*A. baumannii* allelic exchange vector with *sacB* (pFLp2)	Herbert Schweizer (Hoang *et al*., 1998) [76]
Escherichia coli HB101 pRK2013	Helper plasmid for the mobilization of non-self-transmissible plasmids	(Figurski and Helinski, 1979) [77]
Escherichia coli pKNOCK-mTn7(amp^R)	mini-Tn7-Amp	(Carruthers *et al*., 2013) [57]
Escherichia coli pTNS2	Tn7 transposase expression	(Choi *et al*., 2005) [78]
Acinetobacter baumannii ATCC 17978VU pKNOCK-mTn7-Amp	WT integration control	This study
Acinetobacter baumannii ATCC 17978VU Δ*migC* pKNOCK-mTn7-Amp	Δ*migC* integration control	This study
Acinetobacter baumannii ATCC 17978VU pKNOCK-*migC*	complementation strain	This study
Escherichia coli pKNOCK-*migC*	complementation vector	This study
Acinetobacter baumannii ATCC 17978VU *attTn7::migC-C71A, C73A, C74A*	*A. baumannii* strain with point mutations in the metal binding domain	This study
Acinetobacter baumannii ATCC 17978VU *attTn7::migC-E99A*	*A. baumannii* strain with mutations in the GTPase domain	This study

Escherichia coli attTn7::migC-gBlock(*C71A C73A C74A*)	*E. coli* strain with gBlock containing mutations in the metal binding domain	This study
Escherichia coli pYDE007	*E. coli* containing plasmid for sgRNA expression.	(Bai *et al.*, 2021) [46]
Escherichia coli pYDE009	Used for movement of *dcas9* into *A. baumannii. Tn*7 element has tetR-tetP-dcas9	(Bai *et al.*, 2021) [46]
Acinetobacter baumannii<br>*ATCC 17978 attTn7::tetR-tetP-dcas9-rrnBT1-T7Te* Gm^r	*A. baumannii* WT strain containing *dcas9*	This study
Acinetobacter baumannii<br>Δ*migC::aphA attTn7::tetR-tetP-dcas9-rrnBT1-T7Te* Gm^r	*A. baumannii* Δ*migC::aphA* strain containing *dcas9*	This study
Acinetobacter baumannii<br>*ATCC 17978 attTn7::tetR-tetP-dcas9-rrnBT1-T7Te* Gm^r +pYDE007	*A. baumannii* WT strain containing *dcas9* with pYDE007<br>(WT *murD* EV)	This study
Acinetobacter baumannii<br>Δ*migC::aphA attTn7::tetR-tetP-dcas9-rrnBT1-T7Te* Gm^r +pYDE007	*A. baumannii* Δ*migC::aphA* strain containing *dcas9* and pYDE007<br>(Δ*migC murD* EV)	This study
Acinetobacter baumannii<br>*ATCC 17978 attTn7::tetR-tetP-dcas9-rrnBT1-T7Te* Gm^r +pYDE007(sgRNA$_{murD}$)	*A. baumannii* WT strain containing *dcas9* with pYDE007 and a *murD* sgRNA (WT *murD* KD)	This study
Acinetobacter baumannii<br>Δ*migC::aphA attTn7::tetR-tetP-dcas9-rrnBT1-T7Te* Gm^r +pYDE007(sgRNA$_{murD}$)	*A. baumannii* Δ*migC::aphA* strain containing *dcas9* and pYDE007 and a *murD* sgRNA<br>(Δ*migC murD* KD)	This study
Escherichia coli DH5α pKNOCK-mTn7-Gm^r-*tetR-P$_{tet}$-murD*	*E. coli* strain containing inducible *murD* vector	This study
Acinetobacter baumannii<br>*ATCC 17978 attTn7::tetR-P$_{tet}$-murD* Gm^r	*A. baumannii* WT strain containing anhydrous tetracycline inducible *murD*	This study
