# Supplementary material for: The zinc metalloprotein MigC impacts cell wall biogenesis through interactions with an essential Mur ligase in Acinetobacter baumannii
Source: PLoS Pathog. 2025 Jun 16;21(6):e1013209. doi: 10.1371/journal.ppat.1013209 (PMC12208494; doi:10.1371/journal.ppat.1013209)
Supplement: S3 Table — (PDF) [file ppat.1013209.s009.pdf]

<u>Supplementary Table 3:</u> Oligonucleotides used in this study.

**Primer Name**	**5’-3’ Sequence**	**Description**
***migC* mutant construction and confirmation**		
*migC*_Up_F	GGTTAAAAAGGATCGATCCTCTAGATTCGTCATACCAATAGCTGTTATTTC	pFLP2 cloning, 5’ flanking region, forward for *migC* KO construct
*migC*_Up_R	TAGTTAGTCAAAGCGGCTCAAATCAACAAG	pFLP2 cloning, 5’ flanking region, Reverse for *migC* KO construct
*migC*_Down_F	GGAATAATGAAGAACATTGAAATACTTCAATATTCTCTATG	pFLP2 cloning, 3’ flanking region, forward for *migC* KO construct
*migC*_Down_R	AAGTTCCTATTCTCTAGGGGGATCCGGGCGTCGGTATGAATATC	pFLP2 cloning, 3’ flanking region, reverse for *migC* KO construct
*migC*_External_F	TGTTTATTTAAGAAAAACTAAAAAAT	To generate unmarked *migC* mutant, forward
*migC*_External_R	TTTAAGCGCCATAGTTAATATGTC	To generate unmarked *migC* mutant, reverse
*migC*_Kan_F	TGAGCCGCTTTGACTAACTAGGAGGAATAAATGG	5’ primer for amplifying *aphA* for *migC* KO construct
*migC*_Kan_R	TCAATGTTCTTCATTATTCCCTCCAGGTAC	3’ primer for amplifying *aphA* for *migC* KO construct
***migC* complementation**		
*migC*_Comp_F	CATGCATGAGCTCACTAGTGGATCCGTGAAACTGATTGCCCAAAAAAC	Cloning into pKNOCK, forward
*migC*_Comp_R	GCAAGGCCTTCGCGAGGTACCTATTGTTCAATTCTGCAAGC	Cloning into pKNOCK, reverse
***migC* GTPase and metal binding mutants**		
*migC*_C71A_C73A_C74A_gBlock	CATGCATGAGCTCACTAGTGGATCCTTTATGGCATCCTTAAATTCATTAAAAAATAAATCAAACCTAA	gBlock for cloning into pKNOCK

	TCGTTCTGACCTACATCGAACA<br>TATAGGGCTGAATGATTAAGCT<br>AGGACACATGCTAAGCCGAGCT<br>TGGGATTACATCAATTAACTTAA<br>CAATCTATTACGCCTATCTTAAC<br>AATATGAAATATAACGTCCGTTT<br>ATGTGCGTCTACTGTAAAATTAA<br>AGCGCTACTTGAGCGTTAAAAA<br>TGTGAAAGAATATTTCGTATTGA<br>GTAAAAAAAAAGCAAGTTAGTTA<br>CGTAAACAAAACACTCTGGCAT<br>GAATTGTGAATGTCCTTAAATAG<br>GATATTTCGTTTAATTATTATTTT<br>TCTAAAGTTAATTTAGATAATTG<br>ATGAGATGCATGTCACATATTTG<br>GTGCTTTAGCCTGATAATTGAAA<br>AGTATATCTTCAAAGTGAAAAAT<br>TATTTTCATGCTACTTAAACAGT<br>GATATACCTTTCTTAGTTTTTAA<br>ACCTTGTTGATTTGAGCCGCTT<br>GTGAAACTGATTGCCCAAAAAA<br>CTGTTCCTACCCATATTATTTCA<br>GGGTTTTTAGGGGCAGGGAAAA<br>CGACATTATTGCAACATTTATTA<br>AGTCAGAAACCGAAAGATGAAG<br>TGTGGGCTGTCTTAATGAATGA<br>ATTTGGGCAAATCGGAGTCGAT<br>CAACAACTCCTACCTCAAGATC<br>AAGGTTATGCAGTCAAAGAACT<br>ATTGGGCGGCGCTTTAGCTGCT<br>AGTAGCCAACTTCCTATGCATAT<br>TGCTTTGGCTCGCTTGTTAAGT<br>GAACAAAAGCCAGACCGACTCT<br>TTATTGAACCTACAGGGCTTGG<br>AGCTCCTTCGCAATTATTTGATC<br>AGTTAACGGAACCACATTGGCA<br>AAACAGCTTAGCTATGCGTGCA<br>CTAGTGACTGTGGTTGATGGTA<br>GTCGTCTGCATGACACCAATTG<br>GGTCAAGCAAAACCTATATGAA<br>GACCAATTAAAAGCTGCACAGA<br>TTGTGGTGGTCTCTCATGCCGA<br>TACCATGACTTTTGAAGATGAAC<br>AGGCACTTGTTGAGCTTAAAGA<br>AGAATACCAACCTTATCAGCAA<br>CAATGGCTAAAGACTGAACATG<br>GTCAACTTGAACTGGCGCAAAT<br>TGATGTGCCTGCGCGTTTAACT<br>GAACGGTCTATACAACCTTTACT<br>TAAGCTGCAAAAGCAAATGACC<br>GAAGCCGAAGTTGTAAAAGAAA	

	TTCACCAACTGCCTTATCATTATGTTGAGACGGCGCAGGGTTATATGGTCGCGGGTTGGAAATTTCCTAAGCGTTGGAAATTTGAGTTTTATGCTTTGCTCGATGTGTTGTGTGCACAACAAGACTGGCTACGTATTAAGGGAATTTTTAATACAGATCAGGGGTGGAAGACCTTTAACTTTAATCCTGAGCAGTTTAACTATAAGTCTGGTGAAGAGGGCATTGATAACCGCATTGAAATGATTAGCCAACATGAACATGACTGGCTGGCATTTGAAACTGAACTATTAGCTTGCAGAATTGAACAATAGGTACCTCGCGAAGGCCTTGC	
migC_E99A_F	ACTCTTTATTGCGCCTACAGGGC	To generate *E99A* point mutant construct in pKNOCK, forward
migC_E99A_R	GCCCTGTAGGCGCAATAAAGAGT	To generate *E99A* point mutant construct in pKNOCK, reverse
***migC* overexpression**		
murD_OE_F	TATCTGGTTGGCCTGCAAGGCCTGTGAAACTGATTGCCCAAAAAAC	Cloning into pKNOCK, forward
murD_OE_R	GAGGAGAAAGGATCTGGTACCTATTGTTCAATTCTGCAAGC	Cloning into pKNOCK, reverse
murD sgRNA		
murD_sgRNA_F	TTACACTAGTGTCGCAGGCTTGGGAATATCGTTTTAGAGCTAGAAATAGCAAG	Amplification of *murD* sgRNA, forward
murD_sgRNA_R	AAGTGGGCCCAAGCTTCAAAAAAAG	Amplification of *murD* sgRNA, reverse
Protein expression for purification		
MigC_pHIS_F	TAACTTTAAGAAGGAGATATACATATGAAACTGATTGCCCAAAAAACTG	Cloning *a1s_0934* into pHIS-Parallel1, forward

MigC_pHIS_R	TCGACGTAGGCCTTTGAATTCCTATTGTTCAATTCTGCAAGCTAATA	Cloning *a1s_0934* into pHIS-Parallel1, reverse
MigC_E99A_pHIS_F	ACTCTTTATTGCGCCTACAGGGCTTG	To generate *E99A* point mutant construct in pHIS-Parallel1, forward
MigC_E99A_pHIS_R	CGGTCTGGCTTTTGTTCAC	To generate *E99A* point mutant construct in pHIS-Parallel1, reverse
MurD_pHIS_F	GTATTTTCAGGGCGCCATGTTAATACAACGTGGTGG	Cloning *a1s_0245* into pHIS-Parallel1, forward
MurD_pHIS_R	CTTTGAATTCCGGATCCATGTTAAACCAACGAATTGACGC	Cloning *a1s_0245* into pHIS-Parallel1, reverse
MurC_pHIS_F	TCAGGGCGCCATGTCTCCAACAACAGCTGCG	Cloning *a1s_3335* into pHIS-Parallel1, forward
MurC_pHIS_R	CCGGATCCATGTTATTTCACATACAAATGGTGCTGTGC	Cloning *a1s_3335* into pHIS-Parallel1, reverse
