# Supplementary material for: The zinc metalloprotein MigC impacts cell wall biogenesis through interactions with an essential Mur ligase in Acinetobacter baumannii
Source: PLoS Pathog. 2025 Jun 16;21(6):e1013209. doi: 10.1371/journal.ppat.1013209 (PMC12208494; doi:10.1371/journal.ppat.1013209)
Supplement: Attachment — Submitted filename: Critchlow and Rocchio Response to Reviewers 02192025.docx [file ppat.1013209.s011.docx]

**Reviewers’ Comments:**

We would like to thank the reviewers for their insightful and constructive feedback on our manuscript titled, “The Zinc Metalloprotein MigC Impacts Cell Wall Biogenesis through Interactions with an Essential Mur Ligase in *Acinetobacter baumannii*.” Your comments and suggestions have greatly enhanced the quality of this work, and we are grateful for the opportunity to address the points you raised.

We appreciate your recognition of our findings' rigor and novelty, particularly regarding the non-canonical role of MigC in the zinc-dependent regulation of MurD and its implications for cell wall synthesis in *Acinetobacter baumannii*. The thoughtful input provided by each reviewer has been instrumental in refining the manuscript, ensuring its clarity and impact.

Below, we provide detailed responses to the reviewers' comments and outline the revisions made to the manuscript.

**Major Reviews:**

**Reviewer #1**: None noted.

**Reviewer #2**: None noted.

**Reviewer #3**:

**Comment 1:** ***This is quite an interesting piece of work, and the biochemistry is quite cool, but frankly the growth curves are displayed in a way that is totally noninformative. I believe what the authors are saying at a gut level, but I really can’t figure out what is going on when they display logarithmic growth on a linear scale. It makes everything look like a long lag, I really can’t tell whether there are different growth rates or yields are altered, etc. Displaying growth curves in this way is kind of the original sin of bacterial physiology and prevent proper evaluation of the data. This has to be redisplayed properly and then the authors have to study what it says and present the work accordingly, mindful of calculating growth rates (division times) when appropriate.***

**Response:** Thank you for your insightful feedback on the presentation of our growth curves. We believe there has been a misunderstanding based on how we labeled the graphs. To clarify, the growth curves were all graphed on a logarithmic scale, not a linear scale. We recognize, however, that the scale used was not immediately clear due to minimal markings, so we have added intermediate markers and clarified the logarithmic scale in both the figures and the text to enhance readability. In addition, we have calculated and have reported growth rates and maximum density where appropriate, allowing for a more precise interpretation of the data.

A blank correction was applied to all the growth curves, and as a result, the ODs are now at 0.001, which aligns with the expected value from a 1:1000 dilution. Initially, we did not apply the blank correction, which we think caused further confusion. We appreciate your constructive feedback and have made the necessary revisions to improve the clarity and rigor of the data presentation.

As far as the requested quantifications of the growth curves, please find the values below. We thank the reviewer for this critique and appreciate the opportunity to address this directly and have included the values below as well as referenced them in the text in lines 312 and 325 . The following values have been calculated for each growth curve:

**Growth Rate**: The area under the curve of the log-transformed OD vs. time.

**Maximum Density**: The highest OD value for each condition.

**Figure 3A:**

| **Genotype** | **Growth Rate (AUC)** | **Maximum Density** |
| --- | --- | --- |
| **WT *attTn7*: EV LB** | 11.45 | 0.89625 |
| **Δ0934 *attTn7*: EV LB** | 11.01 | 0.8495 |
| **Δ0934 *attTn7*:0934 LB** | 11.16 | 0.868 |
| **WT *attTn7*: EV TPEN** | 2.270 | 0.4155 |
| **Δ0934 *attTn7*: EV TPEN** | .9471 | 0.1715 |
| **Δ0934 *attTn7*:0934 TPEN** | 1.794 | 0.35 |

**Figure 3D:**

| **Genotype** | **Growth Rate (AUC)** | **Maximum Density** |
| --- | --- | --- |
| **WT *attTn7*: EV LB** | 10.64 | .7763 |
| **Δ0934 *attTn7*: EV LB** | 10.51 | .7680 |
| **Δ0934 *attTn7*:0934 LB** | 9.902 | .7135 |
| **WT *attTn7*: EV CP SIIKO** | 5.738 | .4980 |
| **Δ0934 *attTn7*: EV CP SIIKO** | 3.716 | .3640 |
| **Δ0934 *attTn7*:0934 CP SIIKO** | 5.164 | .4500 |

**Figure 5A:**

| **Genotype** | **Growth Rate (AUC)** | **Maximum Density** |
| --- | --- | --- |
| **WT *murD KD* LB** | 6.161 | .5643 |
| **WT *murD KD* 25 ng/mL AhTc** | 5.648 | .6770 |
| **WT *murD KD* LB 50 ng/mL AhTc** | 4.436 | .7163 |
| **WT *murD KD 75* ng/mL AhTc** | 1.417 | .4590 |

**Figure 5B:**

| **Genotype** | **Growth Rate (AUC)** | **Maximum Density** |
| --- | --- | --- |
| **Δ0934 *murD KD* LB** | 7.802 | .5557 |
| **Δ0934 *murD KD* 25 ng/mL AhTc** | 8.370 | .5910 |
| **Δ0934 *murD KD* LB 50 ng/mL AhTc** | 8.581 | .6067 |
| **Δ0934 *murD KD 75* ng/mL AhTc** | 7.301 | .5430 |

**Figure 5D:**

| **Genotype** | **Growth Rate (AUC)** | **Maximum Density** |
| --- | --- | --- |
| **WT *murD KD +*TPEN** | 8.337 | .6447 |
| **Δ0934 *murD KD +*TPEN** | 1.512 | .2517 |
| **WT *murD KD +*AhTc + TPEN** | 5.298 | .4020 |
| **Δ0934 *murD KD +*AhTc + TPEN** | 3.620 | .4317 |

**Comment 2:** ***My second point is the MurD overexpressor data (Fig. 5G)is not particularly convincing (as far as I can tell based on linear growth curves) because the proper controls were not used, and the particular control used (Supp. Fig. 4F?) indicates that ATC addition causes a defect in fitness. I think the simplest solution is to delete the experiment, but if they want to keep the experiment, the control should be an active site mutation in MurD. Furthermore, the control is an “integration control.” I think this is lab jargon, because I have no idea what this means. I suppose it means no dCAS9 at the Tn7 site, but I am not sure. Finally, showing a critical control in Supplement and the experiment in the main text is not appropriate. The experiment only makes sense if you can compare it to control, and it’s a little difficult to flip between figures to determine if the experiment makes sense.***

Thank you for your thoughtful feedback. Based on the Reviewer’s suggestion, we have removed the MurD overexpression data (Fig. 5G). We appreciate your suggestion and agree that this data does not add value to the paper under the current experimental design.

Regarding the "integration control," we used this term to refer to an empty vector backbone inserted into the chromosome, instead of the anhydrotetracycline (AhTc) promoter driving *murD* expression. We understand that this might be uncommon terminology and have ensured this is clearly defined in the revised manuscript.

Lastly, we agree that presenting both the critical control and the experiment in different figures was not ideal. To address this, we have opted to remove the figure panel showing this experiment altogether to avoid confusion and ensure the presentation remains clear and coherent.

We believe these changes significantly improve the clarity and rigor of the study.

**Comment 3: *Although this is speculation, it seems like invoking MigC as an inhibitor is certainly stating what the data are providing, but one might why an inhibitor itself has to be regulated by GTP levels in the cell and Zn ions. One might wonder whether its role is something other than being an inhibitor, but rather, it might be a specificity determinant that ensures side reactions or inappropriate substrates are not targeted by MurD. An example might be thinking about proofreading functions with DNA polymerases. Proofreading reduces polymerization rates, but it would be inappropriate to call proofreading an inhibitor. Another way to look at this is to recall the effects of Mn on Mg-containing enzymes. Mn can increase reaction rates, increase binding affinities, etc., but at the cost of loss of specificity. Mg might result in lower reaction rates and lower binding affinities but with the reward of increasing substrate specificity. One wonders if there are other interfering reactions that MurD can catalyze that could cause cell wall disruption, and the MigC acts to prevent these side reactions.***

We thank the reviewer for these comments. We did not previously consider that MigC might be a “specificity determinant” of some kind for MurD. We have added text to the discussion along these lines and provide a new Supplemental Figure (**Fig. S6**) that shows that *in vitro* MigC does not change the product profile of MurD; in other words, we see only one product, UMAG, in the presence and absence of Zn-MigC, with its known substrate. This may not be the case in cells, and thus we have added the following to the Discussion (p. 18):

“Close examination of MurD product profiles as resolved by our LC workflow reveals only the single expected product, UMAG, in the presence and absence of MigC, at saturating ATP and D-glucose concentrations (**Fig. S6**); this suggests that the integrity of MurD turnover of UMA is not impacted by MigC. It remains formally possible that MigC influences MurD substrate specificity and product distribution in cells, while also modulating flux through this pathway. It also seems plausible that MigC interacts with other client proteins or pathways and such interactions may contribute to these phenotypes. Both possibilities are currently under investigation.”

**Minor Reviews:**

**Reviewer #1:**

**Comment 1**: ***Does the binding of MurD to zinc-MigC affect the latter’s GTPase activity?***

We thank the reviewer for this comment as we had not previously investigated this. We have provided a new Figure (**Fig. 4D**) showing MurD had no impact on Zn-MigC GTP hydrolysis at protein concentrations where the two proteins interact. Text was modified to convey this important aspect (p. 13).

**Comment 2**: ***The symbols are very difficult to distinguish in the growth curves shown in Figs. 3, 5, and S4. This problem could be overcome by using different colors for each sample in the separate panels.***

Thank you for pointing out the difficulty in distinguishing symbols in Figures 3, 5, and S4. We have revised these figures to use distinct colors for each sample, improving clarity and ease of interpretation.

**Comment 3**: ***Figure 3: Panel A includes the undefined “IC” which should be clarified in the legend. Panel D uses the delta migC nomenclature before the MurD interaction was described at which time the gene was renamed; at this point in the text the delta 0934 term should be retained.***

Thank you for pointing that out. We updated Figure 3A legend to define "IC" as the integration control in the text and the legend. Additionally, we have revised Figure 3D to use the "∆*0934*" nomenclature for consistency before introducing the MigC designation.

**Comment 4:** ***Anhydrotetracycline is incorrectly shown as “anhydrous tetracycline” 4 times in the main text and 7 times in the supplemental section. The text could be simplified and the incorrect version eliminated by using the abbreviation (AhTc) that was defined in line 290. Unfortunately, this abbreviation was replaced with “Anhtet” in lines 321, 323, 507.***

Thank you for bringing this to our attention. We have revised the text to consistently use the correct abbreviation “AhTc” for anhydrotetracycline throughout the main text and supplemental section, including in lines 321,323, and 507.

**Comment 5: *In Figure 5, panels A and B indicate varied concentrations, but the substance being varied is not clear without reading the legend. How long were the cells grown for the images in panel C, or is this not important? The y-axis of panel E (Percent LB) must be corrected since the concentration of LB was not varied.***

Thank you for these important points. In panels A and B, we have clarified the substance being varied (AhTc) in the figure legend to make it immediately clear without needing to reference the legend. For panel C, we have included the growth time for the cells in the figure legend. For panel E, we have revised the y-axis label to accurately reflect the data. Instead of "Percent LB," we corrected it to "Plating efficiency (carb/no carb)" to reflect the experimental conditions properly.

**Minor items**:

***Add a reference for “Gecos CLI” in line 165?***

We have added the appropriate reference for "Gecos CLI" at line 165 to ensure proper citation.

***Add “AlphaFold3” reference in line 169?***

We have added the appropriate reference for "AlphaFold3" at line 169 to ensure proper citation.

***Provide references for the zinc-binding affinities in line 179?***

We have provided references for the binding affinities of both competitor dyes used in this study.

***Thermodynamic constants (such as K) should be italicized throughout the manuscript.***

Thank you for your comment. We have italicized thermodynamic constants as well as kinetic constants throughout.

***Line 203: Do the EXAFS data provide insight into whether the O/N ligand is an imidazole?***

In short, not confidently. We have added the following text (p. 9): “These data do not conclusively distinguish between oxygen (O) and nitrogen (N) ligation by the non-thiolate Zn ligand.”

***Line 224: WT was not defined. The usage for WT and delta 0934 varies between being adjectives and nouns, but consistent terminology should be utilized.***

Thank you for bringing this to our attention. We have defined "WT" (wild type) upon its first usage for clarity. Additionally, we have revised the manuscript to ensure consistent usage of "WT" and "Δ*0934*" as either adjectives or nouns, depending on the context, to improve readability and maintain uniform terminology throughout the text.

***Line 242, use medium, not media.***

We have updated "media" to "medium" in line 242 to ensure proper terminology.

***Line 263: is it residues 55-354 or 53-103?***

Thank you for your comment. To clarify, both residues 53–103 and 55–354 are relevant. The original Y2H experiment demonstrated binding between MigC and MurD residues 53–103, while the one-by-one Y2H experiment showed that the truncated form of MurD (55–354) binds to full-length MigC. Together, these results indicate that MigC interacts with a region of MurD that overlaps with its catalytic domain. We have revised the text to clarify this point.

***Line 329: use medium, not media.***

We have updated "media" to "medium" in line 329 to ensure proper terminology.

***Line 338: fix “but to not the liver”.***

We have corrected the phrase to read properly as "but not to the liver" in line 338.

***Line 340: why not use the CRO abbreviation?***

We agree that using the abbreviation "CRO" for ceftriaxone would improve consistency. We have made the change to use "CRO" in line 340.

***Line 415: change pUK18 to pUC18.***

We have corrected "pUK18" to "pUC18" in line 415.

***Line 475: use medium, not media.***

We have updated "media" to "medium" in line 475 to ensure proper terminology.

**Line 488: replace the lab jargon “log” with exponential.**

We have replaced the term "log" with "exponential" in line 488 to avoid lab jargon and ensure clarity.

***Line 508: replace “Overnights” with “Overnight cultures”.***

We have replaced "Overnights" with "Overnight cultures" in line 508 for clarity and precision.

***Line 523: “AA55-354” could be clarified as “amino acids” followed by the residue numbers. Also, is it 55 or 53?***

Thank you for your comment. We have clarified "AA" as "amino acids" followed by the residue numbers. In this case, it is 55, as it references the one-by-one Y2H experiment (amino acids 55–354), which was performed to confirm the results of the broader Y2H data (amino acids 53–103). We have revised the text for clarity.

***Line 524: How do bacterial “kinetic” growth assays differ from bacterial growth assays?***

The term "kinetic" has been removed for clarity, as there is no substantial difference between bacterial "kinetic" growth assays and standard bacterial growth assays in this context. Both refer to the measurement of bacterial growth over time.

***Line 652-653: change “fit with, corrected” to “fit, with correction”.***

We have revised the text to change “fit with, corrected” to “fit, with correction” in lines 652-653 for improved readability and clarity.

***Line 686: define BSA.***

We have defined BSA (Bovine Serum Albumin) upon its first use in line 686 for clarity.

***Line 1026: The term “AA1-200” in line 1026 was not immediately clear and should be described more fully.***

We have revised the text to fully describe the term "AA1-200" in line 1026, specifying that it refers to the amino acid residues 1–200 of the protein in question.

***Line 1027 should include a reference for Gecos.***

We have added the appropriate reference for Gecos in line 1027.

***Line 1065: use medium, not media.***

We have changed "media" to "medium" in line 1065 to ensure correct terminology.

***Line 1069: change order to TEM rather than ETM?***

We have changed the order to "TEM" rather than "ETM" in line 1069 for consistency with the correct terminology.

***Line 1081: define BSA.***

We have defined BSA (Bovine Serum Albumin) upon its first use in line 686 for clarity.

***Line 1098: replace logarithmic with exponential.***

We have replaced "logarithmic" with "exponential" in line 1098 for clarity.

**Supplementary material minor items:**

**Line 4**: *delete the period so the remainder is part of a sentence.*

We have deleted the period for clarity.

**Line 7-8:** *Please rephrase “conserved characteristic … substitution”.*

Thank you for pointing this out. This has been rephrased to,

“The position of the highly conserved aspartic acid (D) to glutamic acid (E) substitution in the G3 loop (E99) of the protein is identified by the arrow.”

**Line 11**: *A verb is needed in the line beginning “Electron” and the order is more commonly seen as TEM, not ETM.*

Thank you for the feedback. We have made the necessary revisions to increase clarity and precision.

**Lines 29, 30, 32, and 39:** *insert “were” before “grown”, and insert “were grown” in line 36.*

All verbiage has been changed to reflect the past tense form of the verbs.

**Line 44**: *what is meant by “kinetically”?*

The word kinetically was removed for clarity.

**Reviewer #2:**

**Line 154.** ***Just for narrative purposes, could you write a few lines about why A1_0934 in particular was prioritized for further study?***

We agree that clarifying why A1S_0934 was prioritized for further study would strengthen the narrative. In response, we have added the following lines to the text:

"This exclusivity to *Acinetobacter* spp., coupled with the absence of regulation by Zur, suggested that A1S_0934 might represent a lineage-specific adaptation with a distinct role in metal homeostasis or bacterial physiology, warranting its prioritization for further study."

***Line 134, the logic is slightly off. Just because a protein is uncharacterized, does not mean that it can serve roles beyond those that are annotated. Perhaps rephrase to “…largely uncharacterized. We hypothesized that some of these proteins may indeed serve other roles beyond metallochaperone…”***

This is a great point. We have addressed this by rephrasing this statement in the following way.

“We hypothesized that some COG0523 proteins may indeed serve other roles beyond metallochaperone activity.”

***Line 239 – another slight narrative issue. The logical flow from Zn sensitivity of a mutant to assessing cell morphology is unclear. The paper that is cited here seems to indicate some connection between zinc starvation and cell wall synthesis; perhaps this could be mentioned as a rationale for looking at cell shapes? Their previous work on a zinc-dependent peptidoglycan hydrolase could also be mentioned here (if zinc starvation induces an endopeptidase, one may expect morphology changes).***

Thank you for pointing out this narrative issue. We appreciate the opportunity to clarify the rationale for assessing cell morphology in the context of Zn sensitivity. The cited paper indeed establishes a connection between zinc starvation and cell wall synthesis, and we agree that this link should be explicitly mentioned as a justification for examining cell shape phenotypes. Furthermore, our previous work demonstrated that zinc starvation induces the zinc-dependent peptidoglycan hydrolase ZrlA, which could explain why zinc-limited conditions might lead to morphological changes. Specifically, if zinc starvation activates an endopeptidase such as ZrlA, one might anticipate alterations in cell morphology as a downstream effect of increased cell wall cleavage.

We have revised this section to incorporate these connections more explicitly, strengthening the logical flow between Zn sensitivity, cell wall remodeling, and the decision to analyze cell shapes. This addition will provide a clearer context for the experimental rationale and better align the findings with previous work.

***Line 252 – I would tread a little lightly here and not call it cell wall turnover (which suggests dynamic measurements, but what was done here is to measure HADA stain intensity). Also, the data show an increase in fluorescence, which indicates either reduced turnover, or increased synthesis. Perhaps it is safest to just describe the phenotype here and call it “increased HADA signal” instead of “peptidoglycan turnover”***

Thank you for this thoughtful suggestion. To provide a more accurate description, we have revised the text to refer to the phenotype as "increased HADA signal" instead.

***Discussion should mention their previous paper showing that a peptidoglycan hydrolase is upregulated during zinc starvation, which the present data are highly consistent with – perhaps it is necessary to relieve inhibition of MurD, thereby increasing flux into PG synthesis, in order to compensate for increased cleavage by ZrlA? The data in Fig. 3F are consistent with this as well – why is the cell diameter of a ∆0934 strain treated with TPEN increased? After all, MurD inhibition is already reduced due to the mutant, and perhaps what zinc starvation adds is PG hydrolase activity***?

We agree that our current findings align well with our previous work demonstrating the upregulation of the peptidoglycan hydrolase ZrlA during zinc starvation. This connection supports the idea that the derepression of MurD activity in the ∆*0934* strain could serve to increase flux into peptidoglycan synthesis as a compensatory response to counteract enhanced cleavage by ZrlA.

The increased cell diameter observed in the ∆*0934* strain treated with TPEN (Fig. 3F) is indeed intriguing. As you suggest, this phenotype could result from the additive effects of unregulated peptidoglycan hydrolase activity during zinc starvation, coupled with reduced inhibition of MurD in the mutant. This dynamic may lead to an imbalance in cell wall remodeling, resulting in morphological changes. We have incorporated these points into the discussion to better contextualize the relationship between zinc starvation, ZrlA activity, and MurD regulation.

***Fig. 5E – the Y-axis labeling is a bit confusing. Percent LB sounds like you used diluted or concentrated LB. Should this be “Plating efficiency (carb/no carb)” or similar?***

Thank you for pointing this out. We understand how the current labeling could be misleading. The Y-axis has been updated to “Plating efficiency (carb/no carb)” to clarify the data.

**Reviewer #3:** No Notes.
