# Supplementary material for: The zinc metalloprotein MigC impacts cell wall biogenesis through interactions with an essential Mur ligase in Acinetobacter baumannii
Source: PLoS Pathog. 2025 Jun 16;21(6):e1013209. doi: 10.1371/journal.ppat.1013209 (PMC12208494; doi:10.1371/journal.ppat.1013209)
Supplement: Attachment — Submitted filename: Critchlow and Rocchio Response to Reviewers 04212025.docx [file ppat.1013209.s012.docx]

**Reviewers’ Comments:**

We appreciate the Reviewers' additional feedback on our manuscript, *The Zinc Metalloprotein MigC Impacts Cell Wall Biogenesis through Interactions with an Essential Mur Ligase in Acinetobacter baumannii.* Below, we address your comments and outline our responses in this round of revisions. Thank you for your continued support and efforts to strengthen our work.

**Reviewer #1:**

**Comment 1: *The new text added in p. 14 and 15 should be reread and edited to ensure accuracy and clarity. Specifically:
-lines 313-315: This sentence feels like a non-sequitur, since the migC-murD double lesion has not yet been compared to murD. Perhaps move this after the next sentence ending in line 317.
-lines 326-329: states upon TPEN treatment, but if referring to Fig. 5D the data do not include TPEN. Please clarify (perhaps this meant to refer to AhTc)?***

**Response:** Thank you for these important clarifications. We have revised the text on pages 14 and 15 to improve clarity and ensure logical flow. Specifically, we have moved the sentence in lines 313–315 to follow the comparison of *migC-murD* and *murD* alone, ensuring a clearer progression of ideas. Additionally, in lines 326–329, we have corrected the reference to TPEN, as the intended comparison was to AhTc, this was an oversight on our behalf and now has been updated to accurately reflect the data in Figure 5D.

**Comment 2:** ***Regarding Fig. S4:
-Please correct the graph titles in panels S4B and S4C. Legend states sgRNAmurD, but titles state pYDE007.
-Please update S4D labels to indicate the sgRNA used (they all state just pYDE007).***

**Response:** Thank you for catching these inconsistencies. We have corrected the graph titles in panels S4B and S4C to match the legend, replacing *pYDE007*-*murD_sgRNA_*. Additionally, we have updated the labels in panel S4D to specify the *sgRNA* used in each condition.

**Comment 3:** ***Additional items:
-lines 511, 517, 520, 544: replace anhydrous/Anhtet with the correct abbreviation
-lines 1105-1107: states strains were ± 75 ng/mL AhTc and plated on carbenicillin to assess viability, but panel 5E graph axis is labelled plating efficiency (Carb/No Carb). Please clarify what this label means, since it is unclear how carb/no carb was involved, and the role of ± ahTc in the experiment appears to be left out from readout.
-lines 1108, 1155: is 20mM correct for TPEN concentration? Figure S4 panel labels show 20 µM.***

**Response:** Thank you for these important clarifications. We have updated lines 511, 517, 520, and 544 to replace *anhydrous* with the correct abbreviation (AhTc). For lines 1105–1107, we have revised the text to clarify the plating conditions were with AhTc in Figure 5E. Strains were grown with and without AhTc to drive expression of the *dcas9* with and without *migC*. Additionally, we have corrected the TPEN concentration in lines 1108 and 1155 to reflect the correct value of 20 µM, ensuring consistency with Figure S4.

**Reviewer 2:**

**Comment 1:** ***Lines 193 and 1049 suggest that Figure 2D investigates the Kd of MigC for Zn in the presence of GTP by fluorescence intensity (according to line 1041); however, the plot shows absorbance changes when changing the concentration of Cd (which is not mentioned in the text). Fig. 2D is the wrong plot!***

**Response:** Thank you for catching this inconsistency. We have replaced Figure 2 with the correct figure and have ensured text agrees with the data presented.

**Comment 2:** ***Line 207 cites Table S3 as the first supplemental table to be mentioned in the text. Renumbering would be appropriate.***

**Response:** We have renumbered the supplemental tables to ensure that they appear in sequential order as they are first mentioned in the text. Table S3 has been updated accordingly.

**Comment 3:** ***Line 231 mentions "empty vector" which might be the meaning of the undefined "EV" in Figure 3A,C,D. Adding a definition would be useful.***

**Response:** Thank you for this suggestion. We have clarified the meaning of "IC" by explicitly defining it as an “empty integration control (IC)" in the figure legend for Figure 3A, C, and D to ensure consistency and clarity.

**Comment 4: *Lines 253-254 appear to reverse what is shown in Fig. S2A and S2B.***

**Response:** We have carefully reviewed Figures S2A and S2B and confirmed that the text in lines 253–254 was incorrectly describing the data. We have revised these lines to ensure they accurately reflect the results shown in the figure.

**Comment 5:** ***Line 290 uses the incorrect phrase "inhibition rates"; the rate of binding that leads to inhibition was not assessed.***

**Response:** Thank you for this clarification. The text has been revised to refer to “inhibition constants” that were determined from the data collected.

**Comment 6:** ***Line 293 seems to lack "of the" after "affinity".***

**Response:** We have revised line 293 to include "of the" after "affinity," ensuring the sentence is grammatically correct and clearer in meaning.
